# Molecular Insights into Bioactive Interactions Within Protein- and Polysaccharide-Based Colloids: Implications for Stability, Functionality, and Bioavailability

**DOI:** 10.3390/foods15010112

**Published:** 2025-12-30

**Authors:** Humeyra Cavdar Dincturk, Nisa Akkuzu, Deniz Günal-Köroğlu, Asli Can Karaca, Esra Capanoglu

**Affiliations:** Department of Food Engineering, Faculty of Chemical and Metallurgical Engineering, Istanbul Technical University, Istanbul 34469, Türkiye; cavdarh21@itu.edu.tr (H.C.D.); nisakkuzu@itu.edu.tr (N.A.); gunald@itu.edu.tr (D.G.-K.); cankaraca@itu.edu.tr (A.C.K.)

**Keywords:** protein-polyphenol interactions, food colloids, polysaccharides, carotenoids

## Abstract

Although bioactive compounds are associated with various health-promoting effects, their practical application is limited by challenges such as low solubility, stability, and bioaccessibility. Proteins, carbohydrates, and hybrid colloidal systems are developed for various purposes, including the transport, protection, and controlled release of bioactives. These systems form the basis for the development of functional foods. The functionality of colloidal systems is driven by interactions between bioactive compounds and macromolecules. This review describes the characteristics of food colloid delivery systems; focuses on the mechanisms of colloid-bioactive compound interactions, particularly polyphenols and carotenoids; summarizes their impacts on functionality, bioaccessibility, and bioactivity; and provides insights into advanced characterization techniques. The mechanisms of interactions between food colloids and bioactive compounds are based on structural characteristics, which directly affect their functional and bioactive properties. Therefore, focusing on the latest technologies used to investigate these interactions, as well as understanding changes in component properties at both molecular and macroscopic levels, is crucial for designing more tunable and functional products in the near future.

## 1. Introduction

In recent years, bioactive compounds with health-promoting effects, including antioxidant, anti-aging, anti-microbial, and anti-cancer activities, have become a central focus of functional food studies [1,2]. Despite their significant health benefits, the use of bioactive compounds is limited due to issues such as low solubility, stability, and bioaccessibility, especially for compounds such as polyphenols and carotenoids. Therefore, colloidal systems are frequently utilized in transforming these ingredients into edible and durable product forms [3].

Food colloids, including suspensions [4], emulsions [5], foams [6], and gels [7], provide structural integrity and functional attributes within complex food systems. Being composite structures composed of proteins and polysaccharides, they play a critical role in transporting and integrating bioactive compounds including polyphenols and carotenoids into food systems [3]. The integration of these compounds into complex food systems is especially valuable for functional food research, which has gained increasing attention in recent years [7,8]. This is motivated by the desire to benefit from their biological impacts within food systems. Accordingly, it is not only necessary to incorporate these components into the food matrix but also to minimize their susceptibility to various environmental factors such as pH, temperature, oxygen, and light [9,10]. Simply the incorporation and delivery of bioactive compounds into colloidal systems offers a very narrow perspective. Beyond the presence of bioactive compounds in colloidal systems, it is essential to examine their functionalities. Moreover, the changes in textural and structural properties resulting from these modifications are critical determinants of the final product quality during food application [11]. These expectations are reflected in studies investigating chemical interactions between macromolecules forming colloidal systems and bioactive compounds. Therefore, understanding how colloidal systems interact with bioactive compounds at the molecular level is required to guide formulation design, enhance product functionality, and design food systems with improved quality attributes.

Previous studies have primarily focused on the delivery of bioactive compounds within colloidal delivery systems and characterization of their physical properties [12,13,14,15]. In addition, several studies have examined the mechanisms by which emulsion systems transport bioactive compounds and influence their bioactivity [16,17,18]. While these studies are valuable in emphasizing the practical importance of delivery systems, they do not focus on the molecular mechanisms of interactions among the structural units forming colloidal systems. Although interactions between polyphenols and carotenoids with colloids have been studied in terms of covalent and non-covalent interactions, these studies do not provide a holistic view of the associated molecular structural changes and modifications. Current studies mainly emphasize the transport and release of bioactives, but rarely address how these colloidal delivery systems depend on the molecular interactions between bioactive compounds and structural matrices such as proteins and polysaccharides.

The predominance of protein-polyphenol interactions in the literature largely reflects the structural compatibility between polyphenolic compounds and protein matrices, which enables diverse binding mechanisms and pronounced effects on functional and biological properties [19]. In contrast, carotenoids, due to their highly hydrophobic nature, are more commonly investigated in association with polysaccharide-based colloidal systems that provide physical entrapment and stabilization [20]. Consequently, the following section focuses on polysaccharide–carotenoid interactions as a complementary model system, reflecting the most extensively reported and representative combinations in food colloid research.

This review offers a comprehensive framework linking molecular interactions and discusses their impacts on functional and bioactive properties. Additionally, the review highlights molecular characterization techniques, including spectroscopic methods, molecular docking, and computational modeling, which are collectively addressed in a dedicated subsection. The focus on molecular-level changes, evolving characteristics, and analytical techniques enables bridging molecular interactions with macroscopic functionality, thereby contributing to the design of sustainable, innovative colloidal systems incorporating bioactive compounds.

## 2. Fundamentals of Colloid-Bioactive Compound Interactions

Colloidal systems used in the preservation, transport, and controlled release of bioactive components are formed by macromolecules such as proteins, polysaccharides, and lipids [12,21]. Protein- and polysaccharide-based colloidal systems are widely studied due to their unique functional properties when interacting with bioactive components. Various lipid-based colloidal systems, such as emulsions, solid lipid nanoparticles, and liposomes, are also investigated for encapsulation of hydrophobic bioactive compounds. These lipid-based systems are mostly stabilized by proteins and polysaccharides, which can influence their interfacial behavior, stability, and digestion characteristics. Therefore, focusing on protein- and polysaccharide-based colloidal interactions is critical for understanding the interactions with bioactive components.

Proteins are macromolecules formed by amino acids linked via peptide bonds. They can be obtained from animal, plant, fungi, algae, and microbial sources. This diversity causes proteins to differ in many aspects, such as amino acid type, sequence, molecular weight (MW), conformation, flexibility, and surface properties, leading to diverse functional characteristics [22]. This significantly influences the behavior of proteins in colloidal systems and their interactions with bioactive components, as structural flexibility is a contributor to the affinity of polyphenols [23]. Polysaccharides are another class of macromolecules composed of monosaccharide units linked by glycosidic bonds. Plant-derived polysaccharides such as starch, maltodextrin, pectin, and gum arabic are widely used in colloidal systems [24,25]. Each monosaccharide type, binding mode, and conformation differs, resulting in distinct properties. Lipid-based colloids are especially effective in transporting fat-soluble bioactive compounds. They are typically used in delivery systems as triglycerides and phospholipids. Triglycerides (triacylglycerols) are esters formed by three fatty acid chains (which may be saturated, e.g., palmitic, stearic acids, or unsaturated, e.g., oleic, linoleic acids) attached to a glycerol backbone. Phospholipids (e.g., phosphatidylcholine or lecithin), unlike triglycerides, are amphiphilic molecules containing two hydrophobic fatty acid chains and a hydrophilic phosphate group. These properties make phospholipids suitable as natural emulsifiers and for stabilizing emulsions [26].

Protein, polysaccharide, and lipid-based colloidal systems can form via self-assembly or be stabilized through covalent or non-covalent interactions [20]. Therefore, it would be an oversimplification to suggest that only one type of interaction dominates the structure. Colloidal systems are often grouped into liposomes, emulsions, hydrogels, microcapsules, micelles, and cyclodextrins, each with unique characteristics [27,28]. Liposomes are colloidal encapsulation techniques using animal- or plant-based phospholipids. Their structures, similar to cell membranes, make liposomes suitable for delivery purposes. Liposomes are used to increase the solubility, bioaccessibility, and controlled release of bioactive components [29]. Emulsions are colloidal systems where water and oil phases are stabilized with amphiphilic molecules. In addition to conventional surfactant-stabilized emulsions, Pickering emulsions stabilized by colloidal particles such as proteins have recently emerged. These can be water-in-oil (W/O) or oil-in-water (O/W) systems. Additionally, double or multiple emulsions (W/O/W and O/W/O) can be used for delivering bioactive compounds [30]. Nanoemulsions are particularly effective in enhancing the bioaccessibility of hydrophobic bioactive compounds due to their rapid digestion under gastrointestinal conditions, owing to their high surface area. Examples include the transport of curcumin [31], quercetin [32], and carotenoids [33] in nanoemulsion systems. Solid lipid nanoparticles (SLNs) are systems that contain small lipid particles dispersed in an aqueous environment and coated with an emulsifier, and are in a crystalline state similar to nanoemulsions, with a water-in-oil structure [34]. Hydrogels are viscoelastic three-dimensional systems containing high amounts of water, enabling the transport of bioactive components. These systems are stabilized by various proteins and polysaccharides, such as gelatin [35], casein [36], and pectin [37]. Often, they involve networks of different particles. Microcapsules, which are formed spontaneously through electrostatic interactions, are important colloidal systems. Mainly, positively charged proteins (e.g., β-lactoglobulin) and negatively charged polysaccharides (e.g., gum arabic) interact with each other, forming systems with high transport, encapsulation, and release rates via coacervation [38,39]. Layer-by-layer (LbL) assembly is another electrostatic-based colloidal application used to create multilayer microcapsules [38]. Cyclodextrins are ring-shaped oligosaccharides composed of glucose units, such as α-, β-, and γ-CD. Due to their unique structure and hydrophobic internal cavity, they can be utilized for transporting and releasing various vitamins, carotenoids, and phenolics [39].

**Figure 1:**
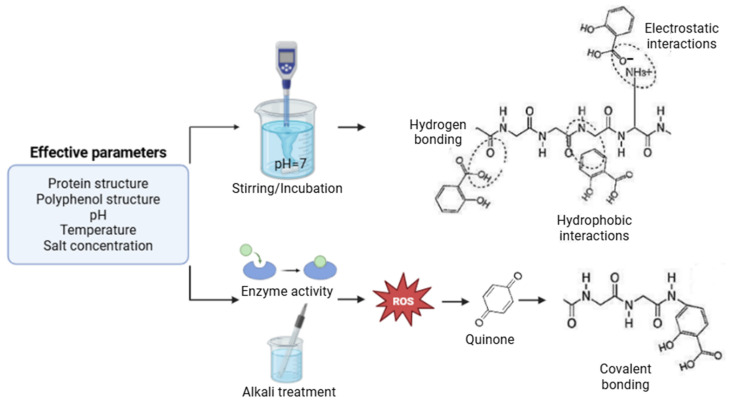
Key parameters influencing covalent and non-covalent protein-polyphenol interactions and their effects on interaction pathways. The arrow represents possible interaction types in complexes, providing a conceptual view of the molecular-level changes occurring during interaction formation [46,47].

The characteristic features specific to colloids and bioactive compounds, as well as their relative ratios, can also govern potential interactions. The molecular weight of proteins, their conformational structure, amino acid profile, and surface properties, such as hydrophobicity, are of critical importance for interactions [40,41]. Larger proteins tend to exhibit higher probabilities of interaction, whereas those with a compact structure may act as barriers to interaction; they show weak features of certain groups [42]. In a food matrix, proteins containing basic amino acids (lysine, arginine, histidine), being positively charged, can engage in ionic interactions with the negatively charged hydroxyl groups of polyphenols. Additionally, the presence of hydrophobic amino acids increases the surface hydrophobicity of the proteins, which underpins hydrophobic interactions with polyphenols [41]. External factors that influence protein-polyphenol interactions include temperature, pH, and the presence of various reagents, which can be listed as environmental factors. Temperature can cause changes in the structural properties of proteins, thereby affecting polyphenol interactions. Since protein denaturation is a process significantly influenced by temperature, it can have both positive and negative effects on these interactions. As a result of denaturation, buried hydrophobic amino acids become more accessible, facilitating hydrophobic interactions with polyphenols. On the other hand, excessive denaturation can lead to protein aggregation, negatively impacting properties such as solubility and thereby hindering interactions. Additionally, high temperatures can directly damage polyphenols [13,43]. Another external factor, pH, causes structural changes in both proteins and polyphenols similar to temperature. Some interactions are stable at acidic pH and show weak affinity at neutral pH. At this point, as pH increases, the tertiary structure of proteins changes, and binding sites may become less available. pH-Shifting colloid-bioactive compound interactions emerge as an effective parameter. Transitioning from alkaline pH to neutral pH can cause proteins to denature and partially unfold, leading to increased hydrophobic interactions [44]. Furthermore, polyphenols develop different ionization properties. Changes in salt concentration can lead to different outcomes depending on the types of polyphenols and proteins involved. The presence and type of salt ions in the environment are also considered influential parameters on these interactions. Specifically, magnesium ions (Mg^2+^) form weak electrostatic bonds with negatively charged regions of bovine serum albumin and enhance the binding affinity of tannic acid (TA) to proteins. Calcium ions (Ca^2+^), on the other hand, show high affinity with the galloyl groups of TA, thereby making hydrophobic interactions with proteins more dominant, whereas manganese ions (Mn^2+^) coordinate with BSA through imidazole groups in a chelation-like manner and, due to their redox properties, promote more hydrophobic binding [45]. Figure 1 represents the key parameters involved in the formation of covalent and non-covalent interactions.

## 3. Protein-Polyphenol Interactions

Proteins and polyphenols interact through two distinct mechanisms: covalent and non-covalent interactions. Regarding reversibility, it can be associated with the fact that non-covalent interactions, such as hydrogen bonds, hydrophobic interactions, van der Waals forces, and ionic bonds, are relatively weaker compared to covalent bonds [40,46]. In non-polar interactions, polyphenols primarily act as hydrogen donors and form hydrogen bonds with the carboxyl groups of proteins. Also, hydroxyl (-OH) groups of phenolics may interact with hydrogen (-OH) or amino groups of proteins [47]. Apart from hydrogen bonding, non-polar aromatic rings of polyphenols can interact with the hydrophobic amino acids of proteins and form hydrophobic interactions. Moreover, electrostatic interactions may occur between the negatively charged hydroxyl groups of phenolics and charged functional groups in the side chains of proteins. Finally, van der Waals forces arising from dipole–dipole attractions by the surrounding solvent on atoms further enhance these non-covalent interactions [48]. For instance, blackcurrant polyphenols interacted with milk proteins mainly through non-covalent interactions, including hydrophobic interactions and hydrogen bonds [49]. Covalent interactions, such as alkaline reactions and free radical grafting, can occur through non-enzymatic conjugations or enzymatic pathways, including phenolic oxidases, such as laccase. In both types of reactions, polyphenols rapidly oxidize and transform into semiquinone and quinone structures, creating crosslinking with nucleophilic amino acid residues (i.e., Met, Lys, Try, and Cys) [50,51].

Hydrophobic interactions typically occur between nonpolar aromatic rings in polyphenols and hydrophobic side groups (e.g., leucine, isoleucine, valine, phenylalanine, and tryptophan) in proteins [47]. Additionally, charged polyphenols can electrostatically interact with proteins, and generally, non-covalent interactions are supported by van der Waals attractive forces. The presence of these interactions significantly influences the various properties of proteins and polyphenols. Covalent conjugation, unlike non-covalent interactions, can occur through chemical methods such as Maillard reactions, free-radical grafting, and alkaline reactions, often catalyzed by enzymes including polyphenoloxidase, laccase, and tyrosinase. Phenolic compounds can interact with proteins via phenolic radicals or quinones [52,53].

### 3.1. Impacts of Polyphenol Interactions on the Structure and Functionality of Proteins

#### 3.1.1. Conformational Alterations

Interactions between proteins and polyphenols include reversible non-covalent interactions and irreversible covalent interactions involving strong C-N and C-S bonds [54]. These interactions result in conformational changes in the secondary and tertiary structures of the proteins. Fourier Transform Infrared (FTIR) spectroscopy, fluorescence spectroscopy, Circular Dichroism (CD) spectroscopy, and molecular docking simulations are used for characterizing the structural changes and binding mechanisms resulting from protein-polyphenol interactions at the molecular level [55]. Each technique provides a unique perspective on these interactions and is analyzed in detail to elucidate the changes arising from the sources of the compounds.

Various studies in the literature examine structural changes in proteins and focus on the functional effects of these changes [23,56,57]. The non-covalent binding of rutin to β-casein did not cause a significant change in the protein’s secondary structure according to CD and FTIR data results; however, molecular docking and fluorescence quenching showed that the tertiary structure was altered. This observation was determined by the proximity of rutin to the tryptophans and proline-rich regions of β-casein [49]. In contrast, it has been emphasized that the covalent interaction of chickpea protein isolate with epigallocatechin gallate (EGCG) results in changes in the secondary structures of the proteins, with an increase in α-helix content and a decrease in β-sheet content [58]. In another study, myofibrillar protein (MP) extracted from *Coregonus peled* fish muscle interacted with four polyphenols: chlorogenic acid (CA) and gallic acid (GA), as well as epicatechin gallate (ECG) and EGCG. It was found that hydrogen bonds are the dominant type of interaction, with an emphasis also placed on hydrophobic interactions. Covalent bonds formed through oxidation at high concentrations and neutral pH were associated with a decrease in free sulfhydryl groups. The CD results also indicated that the protein denaturation involved the transformation of α-helix structures into β-sheet structures [59]. Similarly, it has been stated that covalent crosslinking of soy protein isolate with CA alters the secondary structure of the protein and causes a looser tertiary structure, with tyrosine and tryptophan residues being exposed to the hydrophilic environment [60]. In a study comparing whey protein to plant-based proteins such as pea and hemp, it was reported that the compact globular structure of β-lactoglobulin results in lower binding affinity to whey protein compared to pea and hemp proteins, due to the peptide binding regions being less accessible. The high α-helix and β-turn content in plant proteins was emphasized for its effect on flexibility and binding capacity [61].

Several studies have shown that interactions between proteins and polyphenols depend on the structural properties of the phenolics and the pH at which the interaction occurs. The complexity of protein-polyphenol interactions and their lack of a standard formation present significant research potential regarding how much they are affected by the structural features of proteins.

#### 3.1.2. Functional Properties

Proteins exhibit a range of functional properties in food systems due to their structural characteristics, which can be summarized as solubility, gelling, foaming, and emulsification. These functional properties can be modified through interactions with polyphenols, and the type of interaction can lead to distinct functional outcomes [13]. Table 1 presents the key findings of recent studies on the covalent and non-covalent interactions of proteins and polyphenols.

Protein-polyphenol interactions notably change the surface characteristics of proteins, impacting their solubility, gel-forming, foaming, and emulsification properties. When non-covalent interactions induce partial unfolding of proteins, surface hydrophobicity and protein solubility appear as the primarily affected properties [67]. Recently, covalent interactions between polyphenols and proteins have been demonstrated by studies as an effective method to increase protein solubility [74,75]. These studies mainly emphasize that the decrease in surface hydrophobicity caused by the interaction of polyphenols with hydrophobic amino acids leads to increased solubility. Consistently, He et al. [65] found that the binding of EGCG and chlorogenic acid (CA) to peanut protein extract (PPE) increased the solubility, attributing to changes in the protein structure caused by the addition of hydroxyl groups from polyphenols during covalent interactions.

Polyphenols reduce the surface tension of proteins, creating more flexible interfacial films; this facilitates the formation of small and durable droplets. Similarly, the foaming stability (FS) properties of proteins can also be enhanced through polyphenol interactions. Study on the covalent interactions of EGCG and CA with pea protein [69] revealed improved foaming properties, suggesting deteriorated protein tertiary structure and increased surface hydrophobicity. Studies examining the non-covalent interactions of polyphenols with proteins have also yielded similar observations comparable to those found in covalent interaction research [62,76]. In a study, conjugates of whey protein isolate (WPI) and four polyphenols (EGCG, quercetin [QC], apigenin [AG], and naringenin [NG]) were prepared through free-radical grafting. The results for polyphenol binding equivalents and content of free amino and sulfhydryl groups, as well as those from sodium dodecyl sulfate–polyacrylamide gel electrophoresis, confirmed the covalent interaction between WPI and the polyphenols. FTIR spectroscopy and fluorescence spectrum analysis identified the potential binding sites of the complexes and determined changes in the protein structure. The particle size distribution and scanning electron microscopy data demonstrated increases in conjugate particle sizes and surface changes in the complexes. The conjugation process significantly increased the antioxidant properties and thermal stabilities of polyphenols, whereas surface hydrophobicity was substantially reduced. WPI-EGCG showed optimal functional properties, followed by WPI-QC, WPI-AG, and WPI-NG. In parallel, Li et al. [72] observed that foam stability significantly increased with bovine lactoferrin and procyanidin interactions compared to pure proteins, with the optimal foamability (186%) and foam stability (65%) values observed at the highest level of procyanidin complexation [72].

Gel forming is another property that is affected by interactions involving compounds that can modify hydrophilic interactions, which are expressions of the entrapment of large amounts of water. In a study examining the effects of covalent crosslinking between soy protein isolate and TA, gel hardness reached its highest value (43.33 g); however, further increases in TA concentration decreased gel hardness due to altered crosslinking [71]. Faber et al. [77] studied the effect of EGCG, GA, and TA non-covalent interactions with pea protein isolate (PPI) on the gel-forming properties of protein. It was revealed that gel-forming properties improved with increasing phenolic concentration. This finding was attributed to increased hydrogen bonding, hydrophilic interactions, and crosslinking bonds along with increasing phenolic concentrations. In addition, it has been emphasized that smaller phenolics providing fewer hydroxyl groups at the same phenolic concentration can be effective in the gel strength value [77].

Regarding emulsifying properties, proteins that interact weakly with polyphenols improve the emulsion activity index (EAI) and emulsion stability index (ESI) through the formation of more stable interfacial layers at the oil–water interface.This has been reported particularly in interactions between apple polyphenols and SPI [58]. Additionally, a study examining the effects of pH on these interactions indicated that at neutral pH, EAI and ESI values showed a significant increase, while at acidic pH, they decreased considerably. This situation is attributed to the modification of the protein structure at neutral pH, leading to an increased ratio of α-helix and β-sheet, and consequently, the formation of hydrogen and hydrophobic interactions. The protein structure became more rigid and adopted a more regular conformation. At acidic pH, the increased particle size was attributed to the reduced ability of particles to migrate and adsorb the water-oil interface [18].

### 3.2. Changes in Biological Properties of Polyphenols

The interactions of polyphenols with macromolecules, especially proteins, within the food matrix have been extensively studied and well characterized in recent years [78,79]. These interactions protect polyphenols against oxidation during gastrointestinal digestion while limiting their release in the digestive tract, thereby reducing their bioaccessibility [80]. Interactions of polyphenols such as blackcurrant, tea polyphenols, EGCG, and CA with plant and animal proteins are among the most frequently investigated [11,36,49,70].

#### 3.2.1. Bioaccessibility During Gastrointestinal Digestion

Although the health-promoting properties of polyphenols are highlighted, these effects cannot be explained solely by measuring their antioxidant, antimicrobial, and anti-cancer effects [8]. Polyphenols can only exhibit these effects if they are absorbed at sufficient levels in the body and incorporated into the system. Their ability to show these effects depends on their bioaccessibility, which is expressed as their release from complex food matrices during digestion and their absorption into the bloodstream [81]. The bioaccessibility of polyphenols can be determined through in vitro and in vivo studies. For in vitro approaches, the most common method is the Caco-2 cell monolayer model, which simulates gastrointestinal digestion processes, and it is distinguished from in vivo studies by its implementation in different versions, whether static or dynamic [82].

Interactions between proteins and polyphenols significantly influence the behavior of polyphenols during digestion. In a study in which casein and whey proteins formed covalent interactions with sea buckthorn polyphenols, although the polyphenols alone exhibited high antioxidant activity, a portion of this activity was retained after complex formation with the proteins. This effect was attributed to the protective role of protein-polyphenol interactions against polyphenol oxidation. Additionally, in vitro digestion studies showed that, polyphenols interacting with casein and whey protein exhibited significantly higher bioaccessibility values (60.97–64.97%) than the free polyphenolic extracts (40.01%). Furthermore, in casein-based complexes, encapsulation of polyphenols within micellar structures resulted in lower release compared to whey protein-polyphenol complexes [36]. Similarly, after covalent interactions of milk proteins with blackcurrant polyphenols, antioxidant activity increased by 10–15% and in vitro bioaccessibility by 25–30% [49].

In addition to expressing the quantitative degree of bioaccessibility of polyphenols, how colloidal protein-polyphenol systems respond during the digestion process is also critical. Overall, the nature of protein-polyphenol interactions and the properties of the colloidal matrix strongly influence bioaccessibility. Covalent interactions result in slower release due to the strong retention of polyphenols within the protein matrix against environmental factors. Conversely, non-covalent interactions, which involve weak bonds such as hydrophobic and hydrogen bonds, tend to lead to faster polyphenol release and higher bioaccessibility due to their lower resistance to enzymatic activity and environmental pH. Furthermore, the surface properties of colloidal complexes and the charge distribution, which can be expressed through zeta potential and hydrogen bonding tendencies, increase their interaction with the intestinal mucus layer, leading to effective mucosal adhesion. Colloidal protein-polyphenol interactions play an important role in regulating intercellular transport across the intestinal epithelium [81,83].

#### 3.2.2. Antioxidant Activity

Antioxidant activity is among the most extensively studied bioactivities of polyphenols, owing to their ability to donate electrons or hydrogen atoms and scavenge reactive oxygen species. It can be defined as neutralizing reactive oxygen species (ROS) through hydroxyl groups (-OH) or preventing free radical formation by chelating metal ions [84].

Recent studies have extensively examined how covalent and non-covalent protein-polyphenol interactions influence antioxidant activity. Complexes or conjugates formed through protein-polyphenol interactions are reported to exhibit antioxidant activity similar to that of the polyphenol itself, but at a lower level. In various studies, the decreasing antioxidant activity values are attributed to the protein hindrance effect, which prevents the radical scavenging potential [11,13,49]. In a study examining the interaction of whey proteins (WP) with EGCG and caffeic acid (CA) at different pH levels, it was found that the Folin–Ciocalteu Reducing Capacity and Oxygen Radical Absorbance Capacity (ORAC) values were higher for both phenolics at pH 3.5. Conversely, for Ferric Reducing Antioxidant Power (FRAP) values, the complexes with WP-CA were higher at pH 7, while those with WP-EGCG were higher at pH 3.5. This finding was attributed to the increased Fe^2+^ chelating capacity of CA due to deprotonation of its hydroxyl groups and the potential degradation of EGCG at pH 7. Additionally, it was noted that the interactions are stabilized through non-covalent forces such as hydrophobic interactions and hydrogen bonding. Also, higher antioxidant activity in EGCG was correlated with the high content of phenolic hydroxyl groups [73]. Geng et al. [70] investigated the impacts of ultrasonication on the interactions between soy protein isolate and EGCG. Results revealed that the DPPH (84.84 ± 1.34%) and ABTS (88.89 ± 1.23%) values reached their highest levels compared to the native protein. It has also been stated that the ultrasonic process facilitates the covalent binding of EGCG to the protein, reduces the molecular weight, and modifies the protein structure by decreasing the α-helix content [70]. It has been demonstrated that interactions occurring under different pH conditions can show varying effects on the antioxidant activity of polyphenols. Tea polyphenol-egg white protein interactions were found to be more stable at neutral pH, characterized by high binding affinity and antioxidant activity, and were distinct from those at acidic pH [11].

As described above, various methods are used to evaluate the antioxidant activity of polyphenol-protein complexes. It should also be noted that the antioxidant activity of the protein-polyphenol complexes needs to be compared with that of free proteins and phenolics. The primary reason is that covalent and non-covalent interactions lead to a slight decrease in radical scavenging capacity compared to free polyphenols, due to the steric hindrance effect imposed by proteins. Additionally, the high stability shown towards environmental conditions and the controlled release properties occurring in the digestive system can balance this decrease. When all these findings are considered together, the protection-release balance determined by the interaction type and colloidal organization is critical in determining the biological performance of protein-polyphenol systems, such as their antioxidant activity. Protein-polyphenol and polysaccharide–carotenoid interactions are most frequently described as pairs of biologically active substances and colloids reported in the literature. This pairing is largely due to their complementary physicochemical properties. While different mechanisms are active in the interaction of polyphenols with proteins, the high hydrophobic nature of carotenoids makes them suitable for polysaccharide-based colloidal systems. Therefore, these pairs serve as example models in the relevant literature to demonstrate interaction principles, whereas other combinations have been comparatively less studied.

## 4. Polysaccharide–Carotenoid Interactions

Carotenoids are lipophilic bioactive compounds that show antioxidant activity, provitamin A function, and health-promoting properties. Their application in foods, nutraceuticals, and pharmaceuticals is limited by low water solubility, chemical instability, and sensitivity to heat, light, and oxygen [85]. To overcome these challenges, carotenoids are commonly incorporated into colloidal delivery systems such as emulsions, nanoparticles, nanogels, and inclusion complexes, which protect them from environmental stress and enhance bioaccessibility [86,87].

Polysaccharides serve as versatile carriers in these systems due to their biocompatibility, tunable structures, and ability to form gels, films, or multilayer coatings, stabilizing carotenoids through hydrogen bonding, hydrophobic interactions, and electrostatic forces [21,88]. Structural attributes such as molecular weight, branching, charge density, hydrophilic-hydrophobic balance, and chain conformation critically govern polysaccharide–carotenoid interactions, influencing encapsulation efficiency, colloidal stability, and bioaccessibility [88,89,90]. Chemical modifications of polysaccharides, such as esterification, octenyl succinylation of starch, or TEMPO (2,2,6,6-Tetramethylpiperidine-1-oxyl)-mediated oxidation of cellulose, allow tuning of interfacial properties, molecular conformation, and self-assembly behavior, thereby enhancing the performance of polysaccharide carriers across diverse carotenoid delivery systems [88,91,92,93].

Native starches, primarily composed of amylose and amylopectin, exhibit limited emulsification and encapsulation capacity due to their hydrophilic nature and granular morphology. This inherent limitation highlights the need for chemical modifications to improve their amphiphilic character, enabling better stabilization of hydrophobic bioactives (Table 2) [94]. For instance, octenyl succinylation imparts amphiphilicity, allowing starch molecules to self-assemble into colloidal aggregates with distinct hydrophobic and hydrophilic domains. Wu et al. [88] demonstrated that octenylsuccinated *Gastrodia elata* starch formed stable spherical aggregates with enhanced β-carotene and curcumin solubility. Spectroscopic analyses revealed that β-carotene localized in the hydrophobic core, while curcumin resided near the periphery, stabilized through hydrogen bonding and hydrophobic interactions. In vitro digestion studies showed sustained release, illustrating the role of structural confinement and intermolecular interactions in controlled delivery.

Combining modified starch with other polysaccharides can further improve carotenoid stability. Zhang et al. [92] reported that wet-milling octenyl succinylated starch with β-carotene prior to tea saponin addition produced nanosuspensions with optimized particle size, zeta potential, encapsulation efficiency, and loading capacity. These octenyl succinylated-starch and tea saponin complexes reduced β-carotene crystallinity, preventing recrystallization during storage, and improved dispersion, rehydration, and digestion stability. Microscopy confirmed uniform microcapsule structures with dense polysaccharide coverage, while in vitro digestion revealed enhanced bioaccessibility due to steric and electrostatic stabilization.

Native amylose chains can form V-type inclusion complexes with hydrophobic carotenoids. Bahrololoumi et al. [89] showed that V-amylose (DP 311) complexed with canthaxanthin achieved ~90% recovery, transforming carotenoids into amorphous states, which enhanced antioxidant protection and stability. Ultrasonication (2–10 min, 200 W, 20 kHz) reduced complex size (~244 nm), improving dispersion uniformity and radical scavenging activity, emphasizing the importance of amylose conformation and crystallinity in carotenoid encapsulation and release. Cyclodextrins (α-, β-, and γ-) similarly provide hydrophobic cavities that trap carotenoids via van der Waals and inclusion interactions, enhancing solubility, photostability, and bioaccessibility.

Pectin, an anionic polysaccharide, serves as an effective matrix for the stabilization and controlled release of lipophilic carotenoids through its capacity to form viscoelastic and ionically crosslinked networks. The degree of methoxylation dictates its gelation mechanism and interfacial behavior, enabling both electrostatic and hydrophobic interactions with lipid phases [92]. Modified pectin (DM: 52%) emulsions can produce fine, negatively charged droplets (size: <1 μm, ζ: −25 to −27 mV) with high encapsulation efficiency, particularly when structured with long-chain triglycerides such as olive oil, which enhance interfacial elasticity and carotenoid retention [24]. Han et al. [33] showed that adding β-carotene to the mix slightly lowered the zeta potential due to the interactions at the interface. Olive oil-based emulsions showed higher viscosity and encapsulation efficiency (~87%), attributed to monounsaturated triglycerides enhancing network elasticity. During digestion, pectin matrices preserve emulsion integrity under gastric conditions while modulating lipid hydrolysis and carotenoid bioaccessibility (29–63%), highlighting the relationship between pectin structure and oil composition in carotenoid micellization. Similarly, Mohan et al. [95] reported that pectin concentration, oil content, and homogenization speed significantly affected droplet size and creaming stability in red palm oil Pickering emulsions. Pectin formed a natural shell around droplets, providing surfactant-free stabilization, gel-like viscoelasticity, and protection against oxidation. Thermal and FTIR analyses confirmed physical stabilization without chemical modification, and β-carotene bioaccessibility increased to 37.8% compared to 8.7% in unencapsulated oil, demonstrating the efficacy of pectin networks in enhancing solubilization and digestive release.

Alginate, a linear copolymer of β-D-mannuronic (M) and α-L-guluronic (G) acids, has become one of the most versatile polysaccharides for encapsulating and delivering carotenoids due to its ability to form ionically crosslinked networks with calcium ions (Ca^2+^). This Ca-alginate system relies on the “egg-box” model, where Ca^2+^ bridges the carboxylate groups of G-blocks, yielding hydrogels with tunable porosity, elasticity, and diffusion behavior [100]. The mannuronic-to-guluronic acid ratio (M/G ratio) and crosslinking density affect the mechanical strength and permeability of the gel, thereby controlling the entrapment efficiency and release kinetics of lipophilic bioactives [96]. Encapsulation of carotenoid-enriched oils in Ca-alginate beads achieves remarkably high encapsulation efficiencies, typically between 82% and 98%, as reported across various systems [96,97,98]. This efficiency stems from the mild gelation conditions that preserve carotenoid integrity and antioxidant activity while minimizing oxidative and thermal degradation [97,98]. The hydrophilic alginate matrix acts as a semi-permeable barrier that restricts oxygen diffusion, maintains uniform oil dispersion, and prevents phase separation, thus stabilizing sensitive carotenoids in amorphous, bioavailable forms.

The physicochemical properties of alginate hydrogels also enable controlled, site-specific release in the gastrointestinal tract. Their pH-responsive behavior, shrinking under gastric acidity and swelling in intestinal pH, allows minimal release in the stomach and gradual diffusion in the intestine, improving micellization and absorption of carotenoids [97,99]. Increasing Ca^2+^ concentration enhances gel compactness and elasticity, reducing acid-induced swelling and providing finer control over release profiles. Analytical and spectroscopic techniques (FTIR, DSC, SEM, Raman, and confocal microscopy) have elucidated that carotenoids are physically entrapped rather than chemically bound, ensuring biocompatibility and preserving functionality [96,98,99]. These findings indicate that alginate’s molecular conformation, ionic crosslinking capacity, and pH-sensitive swelling behavior make it an functional carrier for carotenoid encapsulation, balancing structural stability with diffusion-controlled release to protect and deliver lipophilic antioxidants efficiently.

Gum polysaccharides, including Arabic, guar, xanthan, gellan, cashew, and basil seed gums, offer versatile platforms for carotenoid stabilization and controlled release due to their structural diversity, which encompasses branching, molecular weight, charge distribution, and hydrophobic moieties (Table 3). These features facilitate emulsification, droplet stabilization, and carotenoid binding through a combination of electrostatic, hydrogen-bonding, and hydrophobic interactions, while simultaneously forming viscoelastic networks that enhance physical stability [21,90,101]. Acetylated cashew gum and fucan-coated variants, for instance, produced polymeric nanoparticles capable of encapsulating lycopene-rich guava extract with preserved antioxidant activity over prolonged storage, demonstrating how chemical modification can synergistically enhance hydrophobic interactions and electrostatic stabilization [101]. Similarly, basil seed gum-based spray-dried nanocapsules achieved high encapsulation efficiency (≈88%) and antioxidant retention (>75%), with application in food matrices such as mayonnaise, reducing lipid oxidation and sensory deterioration, highlighting the functional efficacy of gum-based colloids [87]. Comparative studies with maltodextrin-gum microcapsules indicate that Arabic gum matrices provide superior encapsulation and carotenoid preservation during storage, attributable to the formation of amorphous, oxygen-limiting polysaccharide films [102].

The rheological and structural characteristics of gums can have significant effects on droplet size, emulsion stability, and controlled release profiles. Moderate concentrations of guar gum form weak gel-like emulsions with viscoelastic behavior that slows creaming and protects β-carotene against degradation, whereas highly viscous xanthan systems may promote flocculation and faster carotenoid loss [103]. High-acyl gellan gum reduces droplet size, enhances encapsulation efficiency (~84%), and resists thermal coalescence, providing shear-thinning, stable emulsions suitable for gastrointestinal delivery [87]. Plant-derived polysaccharides with higher molecular weight and arabinose content exhibit denser droplet interfaces, increased zeta potential, and improved colloidal stability, reducing β-carotene degradation and enabling controlled release [90]. Across these systems, analytical and spectroscopic techniques, including UV-Vis, HPLC, DLS, TEM, AFM, FTIR, and rheology, have elucidated the interactions between gum matrices and carotenoids [87,101,102,103], demonstrating that molecular tailoring of polysaccharides can optimize encapsulation efficiency, oxidative protection, and site-specific delivery, establishing gums as highly adaptable carriers in advanced polysaccharide-based colloidal systems.

Cellulose-based polysaccharides, including nanofibrillated cellulose, nanocrystalline cellulose, and TEMPO-oxidized cellulose nanofibers, provide versatile platforms for carotenoid stabilization and controlled release (Table 3) due to their high-aspect-ratio fibrillar structures, which form protective networks around oil droplets in emulsions, establishing steric hindrance and electrostatic interactions that enhance stability [93]. The structural diversity among these celluloses affects emulsion characteristics and digestive behavior in such a way that nanocrystalline cellulose, with short rod-like particles, produces small, uniformly dispersed droplets that enhance β-carotene bioaccessibility, whereas nanofibrillated cellulose forms long, entangled networks that increase viscosity, promote bridging flocculation, and slow lipid digestion, thereby improving carotenoid retention during storage and processing [106,107]. Pickering nanoemulsions stabilized by nanocellulose maintain particle sizes below 250 nm over extended periods, protecting carotenoids from UV and oxidative degradation, with network density and oil fraction critically modulating release kinetics in simulated gastrointestinal conditions [86,108]. TEMPO-oxidized cellulose nanofibers further enhance encapsulation efficiency (~94%) and bioaccessibility (~60%) post-digestion, demonstrating strong interfacial adsorption and network formation around oil droplets, while maintaining structural integrity under thermal and storage stresses [91].

Beyond food applications, cellulose-based polysaccharides are adaptable for pharmaceutical formulations, demonstrated by hydroxypropyl methylcellulose-K4M/Carbomer gels for topical astaxanthin delivery, which achieve rapid release, high antioxidant activity, and favorable rheology, highlighting the potential of fibrillar polysaccharide networks [105]. Across these systems, the relationship between molecular conformation, fibrillar network formation, and electrostatic stabilization affects droplet morphology, emulsion viscosity, and carotenoid retention. Analytical techniques, including DLS, ζ-potential, microscopy, DSC, and in vitro digestion, have elucidated these structure-function relationships, confirming that nanofibrillated cellulose, nanocrystalline cellulose, and TEMPO-oxidized cellulose nanofibers enable precise modulation of encapsulation efficiency, oxidative protection, and controlled release [86,91,106,107,108]. These findings indicate that cellulose-derived colloids can be used as efficient carriers for lipophilic bioactives, due to their fibrillar structure and interfacial interactions to optimize both food and pharmaceutical delivery systems.

Multicomponent polysaccharide systems, combining chitosan, alginate, pectin, and various gums, exploit synergistic structural and physicochemical interactions to optimize carotenoid encapsulation, stabilization, and controlled release (Table 4). Unlike single-polysaccharide matrices, complex systems enable the formation of multilayered networks and dense colloidal matrices that immobilize carotenoids in amorphous states, reduce oxygen and light exposure, and modulate gastrointestinal release. Electrostatic interactions between cationic chitosan and anionic alginate or pectin, hydrogen bonding among hydroxyl and amino groups, and polymer-polymer interfacial adsorption form strong and viscoelastic networks, providing both physical entrapment and chemical protection [109,110,111,112,113,114]. These multilayered colloids transform crystalline carotenoids into amorphous, uniformly distributed phases, as evidenced by SEM, XRD, FTIR, DSC, and TGA analyses, enhancing thermal and photostability while reducing degradation during storage or processing [109,115,116].

Further, incorporation of composite polysaccharide walls or ternary systems allows tuning of network porosity, charge density, and viscoelastic properties, thereby controlling droplet morphology, release kinetics, and bioaccessibility. Chitosan-coated liposomes and alginate-chitosan nanocarriers demonstrate slow, sustained release and improve mucoadhesion, enhancing carotenoid absorption in simulated gastrointestinal models [112,114,118]. Multilayer emulsions with additional polysaccharides such as maltodextrin or guar gum further enhance oxidative protection, stabilize Pickering-type droplets, and delay astaxanthin release, achieving bioaccessibility levels up to ~70% [110,116]. These studies highlight that complex polysaccharide-based colloidal systems allow for advanced carotenoid delivery, surpassing the capabilities of single-polysaccharide formulations through improved mechanical integrity, protective microenvironments, and controlled release functionality.

## 5. Protein–Polysaccharide-Based Hybrid Colloidal Systems

**Figure 2:**
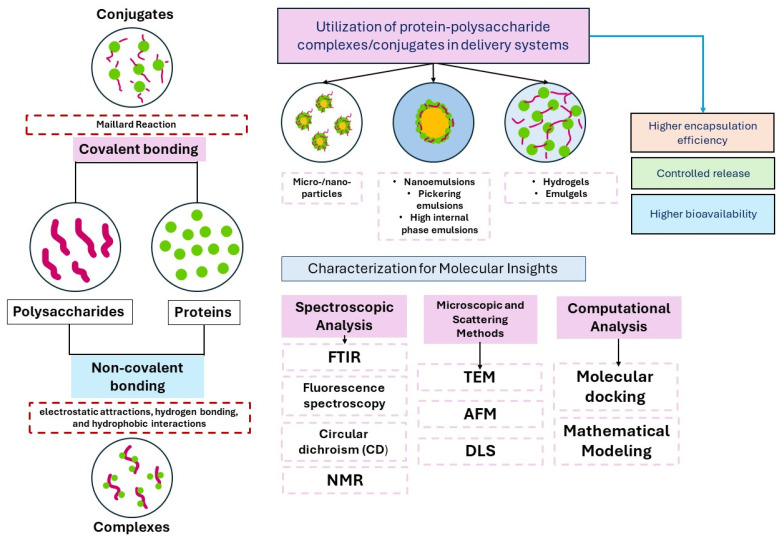
Schematic illustration of protein–polysaccharide interactions and their application in various delivery systems for encapsulating bioactive compounds [124,126,127,128].

Proteins can be derived from animal-based foods (e.g., casein, whey proteins, egg protein, and gelatin) or plant-based foods (e.g., gluten, zein, pea protein, soy protein, quinoa protein, and peanut protein) [119]. The functional properties of proteins, such as emulsifying, foaming, and gelling, enable their application in the delivery of bioactive compounds [120]. However, there are limitations in using proteins alone in encapsulation systems, since proteins exhibit instability due to environmental stimuli during processing, storage, and gastrointestinal digestion [121]. Therefore, protein–polysaccharide composite delivery systems are used to minimize protein precipitation or aggregation by regulating electrostatic repulsion and steric hindrance. Protein–polysaccharide interactions can occur through non-covalent interactions (complexes) or covalent interactions (conjugates). While protein–polysaccharide complexes result from electrostatic attractions, hydrogen bonding, and hydrophobic interactions, conjugates are formed by the Maillard reaction, which includes covalent bonding between the N-glycosylamine group and carbonyl groups of polysaccharides [122]. Up to date, protein–polysaccharide complexes or conjugates have been utilized in the fabrication of a wide variety of carriers, such as micro/nanoparticles, liposomes, hydrogels, emulsions, and emulgels [123,124,125,126,127]. Figure 2 presents the formation and applications of protein–polysaccharide-based delivery systems for encapsulating bioactive compounds. A summary of recent studies on protein–polysaccharide-based encapsulation systems is presented in Table 5.

In a recent study, curcumin was loaded into ovalbumin/polysaccharide (OVA/PS) complexes. The intensification of C-O bond peaks in FTIR spectra of OVA/PS was attributed to the formation of non-covalent bonds between ovalbumin and all polysaccharides (sodium alginate, gum Arabic, and carboxymethyl cellulose). In addition, characteristic peaks of free curcumin at 963.41 cm^−1^, 890.12 cm^−1^, 860.22 cm^−1^, and 812.00 cm^−1^ disappeared in OVA and OVA/PS carriers, potentially due to interaction between curcumin and OVA or OVA/PS. Interactions between OVA/PS and curcumin through hydrogen bonding and hydrophobic interaction provided high encapsulation efficiency (>97%) [131]. The non-covalent interactions between polyphenols and proteins mainly include hydrogen bonding and hydrophobic interactions. While hydrogen bonding occurs between the hydroxyl groups of polyphenols and the carbonyl groups of proteins, hydrophobic interactions occur between the benzene ring of polyphenols and the aliphatic or aromatic amino acids of proteins [132]. In another study, hesperidin was encapsulated in SPI/chitosan, SPI/guar gum, and SPI/xanthan gum complexes. The highest EE% was tested for XG complexes as 90.22%. The UV and fluorescence spectroscopy revealed hydrophobic interactions between XG and SPI. Also, the CD spectrum of SPI and SPI complexes showed a slight decrease in α-helix content and a decrease in the β-sheet of SPI after complexation, which was attributed to primary interaction on the surface of SPI, with minimal influence on its internal conformation [133]. These findings demonstrate that non-covalent interactions, such as hydrogen bonding and hydrophobic interactions, play a crucial role in stabilizing protein–polysaccharide complexes and enhancing the encapsulation and retention of bioactive compounds.

## 6. Advanced Characterization Techniques

### 6.1. Spectroscopic Analysis

FTIR spectroscopy is widely used to identify the functional groups of bioactive compounds and to evaluate potential structural changes or molecular interactions occurring during encapsulation. FTIR enables the characterization of physical or chemical interactions between bioactive compounds and carrier polymers [134]. Shifts in FTIR peaks, along with the disappearance or reduction of characteristic bands, can indicate the entrapment of bioactive compounds within polymer matrices [16]. Recently, curcumin and EGCG were encapsulated in soy protein fibril hydrogels. FTIR analysis revealed that characteristic peaks of free Cur and EGCG disappeared or shifted upon binding to soy protein fibrils, indicating interactions between the protein fibrils and polyphenols. In addition, it was observed that the intensity of protein amide bands changed depending on EGCG concentrations, which was attributed to the conformation of protein secondary structure [135]. In another study, β-carotene was loaded in WPI-GA emulsions, followed by electrospinning. When the FTIR spectra of native WPI and GA and WPI-GA nanofibers were compared, it was revealed that various characteristic bands were displaced, which was linked to the complexation of WPI and GA via hydrogen bonding. Moreover, the main characteristic peak observed in the IR spectrum of free β-carotene appeared in the spectrum of nanofibers, which is an indication of successful entrapment within the fiber [136]. These studies highlight that FTIR analysis can confirm the incorporation of bioactive compounds within the delivery systems. Furthermore, it can enable the identification of types of interactions (e.g., hydrogen bonding, hydrophobic interactions, electrostatic interactions) between carrier polymers and the encapsulated compounds.

Fluorescence spectroscopy measures the intensity and wavelength of emitted light and is commonly used to investigate the molecular interactions between bioactive compounds and carrier materials [137,138]. The reduction of the fluorescence intensity of carrier materials can be an indication of interaction with the core material [135]. The fluorescence intensity of whey protein nanoparticles decreased after loading propolis, which was related to propolis binding to tryptophan residues of WPI [139]. Similarly, when ferulic acid, resveratrol, and rhein were encapsulated in casein nanoparticles, fluorescence intensity declined due to the quenching effect of polyphenols [138]. Moreover, when vitamins C and E were encapsulated in SPI/pectin particles, a noticeable decrease in fluorescence intensity was observed, indicating fluorescence quenching caused by vitamin binding into fluorophore groups in SPI, conformational changes in the SPI, and electrostatic interaction of SPI and pectin [140]. Fluorescence spectroscopy not only provides insights into the interactions between bioactive compounds and wall materials but also helps to understand the molecular interactions occurring among combined wall components, thereby supporting the design of more stable encapsulation systems [141].

Yeast carboxymethyl glucan (YCG) was incorporated in zein nanoparticles to enhance physicochemical stability and more effectively deliver resveratrol. Zein had a fluorescence emission peak at 304 nm, which was attributed to tryptophan residues and tyrosine residues. The incorporation of YCG increased the intensity of tryptophan emission peaks, which was explained by molecular unfolding due to interactions between the hydrophilic groups of zein and YCG [142].

CD is a commonly used technique for analyzing the structures of proteins and polysaccharides by measuring the difference in absorption between left- and right-handed circularly polarized light as it passes through a chiral molecule. It is a widely used tool to investigate the secondary structure of proteins by enabling the determination of α-helix, β-sheet, β-turn, and random coil contents [143]. A study aimed to determine the critical unfolding pH of cod proteins to enhance their binding efficiency with curcumin and improve EE. It was found that at pH 9.5, the spectrum of CP exhibited an apparent blue shift, indicating a decrease in α-helix content and protein unfolding. The critical pH for protein unfolding was determined as pH 10, which provided higher EE%. It was attributed to the formation of smaller protein particles, which exposed more functional groups for curcumin binding [10]. In another attempt, curcumin was encapsulated by casein–polysaccharide (carrageenan, chitosan, and CMC) complexes, and these complexes were characterized using CD. The α-helix, β-sheet, β-turn, and random coil contents of WPI were determined as 7.3%, 39.3%, 21.9%, and 31.6%, respectively. After the incorporation of curcumin, the α-helix content decreased while the β-sheet content increased, which is considered indicative of better protein hydration capacity and more effective entrapment of curcumin [144]. CD was also utilized for investigating the entrapment of EGCG in WPI/pectin particles. It was observed that WPI exhibited decreased β-sheet content and increased α-helix content with increased EGCG concentration, which was linked to stabilization of the α-helix structure by hydrogen bonding between WPI and EGCG [145]. Overall, these studies reveal that CD is a powerful tool for investigating structural changes in proteins upon interaction with bioactive compounds. It can be used to optimize the stability and efficiency of protein-based delivery systems, guiding the development of more efficient encapsulation strategies.

The Nuclear Magnetic Resonance (NMR) technique is based on the subjection of active nuclei by radiofrequency (RF) waves, causing their magnetic moments (spins) to transition between different energy levels. The resulting non-uniform distribution of nuclei in low and high energy states generates a measurable signal, which can be analyzed to provide detailed information on molecular structure, dynamics, and interactions [146]. NMR technology can provide detailed insights into the chemical structure, molecular dynamics, crystallinity, and mobility of encapsulation systems [147]. In a study, curcumin was encapsulated in hydroxypropyl-β-cyclodextrin (HPβCD), and CDs were incorporated in pullulan/OSA nanofibers. NMR was utilized to determine the approximate loading amount of curcumin in nanofibers. The NMR spectra of free curcumin and curcumin-loaded HPβCD showed that the characteristic peaks of curcumin were similar after the incorporation in nanofibers, which indicated the protection of curcumin structure during electrospinning. The loading capacity of curcumin was calculated as 7.8%, and the EE% as 96.9% [148]. Fish oil was encapsulated in layer-by-layer (LBL) lecithin/WPI nanogels. A blueshift of the T2 relaxation time from 114.9 to 103.5 ms was observed after fish oil was encapsulated within LBL nanogels, which was attributed to the decreased proton mobility of the fish oil with the increased glyceryl monostearate (GMS) concentration. This was also observed in thermal stability analysis, which revealed that nanogels with higher GMS exhibited better thermal stability. Also, the transition of crystalline nanogels into an amorphous state indicated the successful incorporation of fish oil in LBL nanogels [149].

### 6.2. Microscopic and Scattering Methods

Transmission Electron Microscopy (TEM) is used for visualizing encapsulation, aggregation, and release mechanisms, comparing the morphologies and distribution in carrier systems. Visualizing with TEM is based on the strong interactions of electrons with proteins, polysaccharides, lipid-based carriers, and other food components, which allow high-resolution images [150]. In the studies focusing on encapsulation of food bioactives, TEM is utilized for observation of the diameter, particle size, and shape of carrier systems. It is also used for the identification of interaction behavior between biopolymers, such as protein-protein and protein-polyphenol binding. Moreover, TEM revealed interactions under specific conditions and insights into the structural stability of encapsulated systems after bioactive incorporation [151,152]. Atomic Force Microscopy (AFM) is another commonly used method that enables nanoscale imaging and measurement of surface morphology and interaction forces, providing detailed insight into the interfacial properties of encapsulation systems [153]. In the study where resveratrol was encapsulated within zein–GA coacervates, AFM analysis revealed that complexation with GA increased the particle size and surface roughness, which was attributed to enhanced aggregation due to electrostatic interactions and chain entanglement. After the ultrasound treatment, the nanoparticles exhibited decreased size, more uniform dispersion, and less surface roughness, which was explained by the disassembly of large aggregates and reformation into smaller spherical particles. These structural modifications likely facilitated stronger binding interactions and improved the encapsulation efficiency of resveratrol [154]. The dynamic light scattering (DLS) technique measures particle size based on the Brownian motion of particles suspended in liquid, where smaller particles move faster than larger ones. In delivery systems, DLS is widely used for observation of size distribution, stability, and aggregation behavior of colloidal systems such as emulsions, liposomes, and nano/microcapsules. Particle size and zeta potential are key parameters influencing EE, release characteristics, and stability of delivery systems, making DLS an essential tool for characterizing carriers [155].

### 6.3. Computational and Integrative Approaches to Colloid-Bioactive Interactions

Computational tools such as mathematical modeling and molecular docking provide insights into colloid–bioactive interactions, enabling prediction of both macroscopic release kinetics and molecular-level interactions [133,156]. Molecular docking is a computational approach that predicts binding sites and simulates the interaction between small molecule ligands and large molecule receptors [157]. Molecular docking can be employed to investigate molecular interactions between carrier materials, as well as carrier-bioactive interactions [130,138]. Molecular docking revealed that cinnamaldehyde was successfully encapsulated within the cavities of β-CD and its modified derivatives. The binding involved hydrogen bonding and hydrophobic interactions, with binding energies ranging from −3.7 to −4.72 kcal/mol, indicating favorable complexation [158]. Three polyphenols (curcumin, quercetin, and resveratrol) were encapsulated in PPI nanocarriers. Molecular docking of 11S legumin with three polyphenols showed that quercetin exhibited the strongest binding affinity, followed by resveratrol and curcumin. All three polyphenols bound in similar sites of legumin with interactions primarily driven by hydrogen bonds, hydrophobic forces, and van der Waals interactions. These docking results were in parallel with fluorescence spectroscopy and FTIR findings, providing a molecular basis for the encapsulation and quenching effects of polyphenols in pea protein [159].

To design effective delivery systems, it is crucial to investigate colloid–bioactive interactions using both computational and experimental techniques, enabling the prediction of interaction, stability, and release behavior. Vitamin E and quercetin were encapsulated in soybean lipophilic protein (SLP)-based nanoparticles, and these nanoparticles were characterized by fluorescence spectroscopy, FTIR, CD, and molecular docking. Fluorescence spectroscopy revealed that pH-shifting combined with ultrasonication treatments changed the structure of soybean lipophilic proteins (SLP), which resulted in protein unfolding and exposing hydrophobic groups. CD and FTIR analyses revealed protein unfolding, including increased β-sheet content and enhanced hydrogen bonding. Moreover, CD and FTIR analyses confirmed complexation of SLP, vitamin E, and quercetin. Molecular docking supported these findings by revealing that vitamin E and quercetin bind to specific pockets on 7S and 11S proteins through hydrophobic interactions, hydrogen bonding, and electrostatic interactions [160]. Overall, this study demonstrates that combining molecular docking with spectroscopic analyses provides a good strategy for studying protein–bioactive complexation at both molecular and macroscopic levels.

It is also evident that understanding the interaction mechanisms of plant-based protein-polyphenol systems at a molecular level is necessary to improve the sensory quality of products. Molecular docking studies have also shown that protein-phenolic interactions are mostly stabilized by reversible non-covalent interactions (hydrogen bonds and van der Waals forces), and that the binding strength varies depending on the phenolic structure, pH conditions, and protein surface properties [76,161,162,163]. These findings demonstrate that molecular docking approaches provide a powerful complementary method for understanding aroma transfer, sensory quality, and interaction mechanisms in plant-based protein systems.

## 7. Effects on Nutrient Delivery and Bioactivity

Microencapsulation and nanoencapsulation techniques can be applied to enhance the stability and bioaccessibility of bioactive compounds. Encapsulation can enable protection of bioactive compounds from environmental conditions during processing, storage, and gastrointestinal digestion and their controlled release in targeted sites by entrapment in a protective matrix [164,165]. The chemical interactions between core and wall materials, as well as between wall components themselves, determine the EE and stability of delivery systems. Protein-based carriers can interact with co-carrier materials such as polysaccharides through hydrophilic or hydrophobic residues. The arrangement of polysaccharide functional groups creates hydrophilic and hydrophobic regions that facilitate polar and non-polar interactions within the encapsulation matrix [165,166]. In a recent study, astaxanthin was encapsulated in octenyl succinic anhydride starch (OSAS)/polyvinyl alcohol (PVA) nanofibers. In TEM images, it was observed that astaxanthin-loaded nanofibers exhibited a core–shell structure with a distinct internal dark area and external bright area. These observations were in parallel with FTIR spectra of astaxanthin-loaded nanofibers, which did not exhibit any characteristic peaks of free astaxanthin. In addition, when the OSAS concentration of astaxanthin fibers was increased, O-H absorption bands exhibited a redshift. It was attributed to the increased hydrogen bonding between OSAS and PVA, which provided more effective protection of astaxanthin. As expected, increased OSAS enhanced the EE% of OSAS/PVA nanofibers. Moreover, increased OSAS concentration provided increased oral release of astaxanthin in simulated saliva, potentially due to a more hydrophilic nature and higher EE% [167]. In another study, liposomes were modified by decanoic acid and stearic acid for the encapsulation of bioactive peptides. FTIR spectra of native liposomes and modified liposomes revealed that fatty acids and liposome membranes exhibited electrostatic interaction, which was an indicator of fatty acid insertion into the bilayer. Also, the incorporation of fatty acids reduced the fluorescence intensity, which was attributed to hydrogen bonding between the alkyl chains of fatty acids and phospholipid chains. However, both native and modified liposomes had low EE%, which was explained by low loading capacity due to the limited aqueous volume of liposomes and diffusion of hydrophilic bioactive peptides into the external aqueous phase. During in vitro digestion, both decanoic acid and stearic acid-modified liposomes showed lower release of bioactive peptides in SGF, potentially due to a more stable and strengthened bilayer structure [168]. These findings highlight that the structural characteristics of carrier materials, whether polymeric matrices or lipid-based systems, directly influence the EE and controlled release of bioactive compounds. Molecular interactions/modifications can enhance carrier functionality. However, these interactions do not always result in enhanced EE and bioaccessibility, since limitations of the utilized method may still cause loss of bioactive compounds.

Encapsulated bioactive compounds are incorporated into food matrices to improve their nutritional value, sensory properties, and functional performance [169,170]. The encapsulation of curcumin in the EGCG matrix did not affect its fluorescence intensity, potentially due to π-π stacking occurring at the benzene rings at both ends of the molecule, which did not affect its enol structure in aqueous solutions. In addition, molecular docking results revealed that curcumin was incorporated into EGCG nanoparticles by π-π stacking interactions between the A-ring and B-ring of EGCG with the benzene ring of curcumin [171]. In another study, after loading caffeine into blank nano-chitosomes, a new peak appeared, and most of the absorption peaks were intensified, which was linked to the interaction between caffeine and the bilayer. The EE of nano-chitosomes varied between 91.2% and 70.71%. During in vitro digestion, nano-liposomes released a total of 98.1% caffeine, while nano-chitosomes released 70.9% [172]. The stability of delivery systems can be influenced by the characteristics of food matrices, such as pH, ionic strength, and the presence of salts, sugars, polysaccharides, and proteins, which may interact with encapsulated compounds. Advanced molecular-level insights into encapsulation systems are essential for improving the gastrointestinal stability and controlled release of bioactive compounds. In addition to the investigations of encapsulated systems, it has been demonstrated that the interactions of aroma-active compounds such as aldehydes with plant proteins mainly occur through hydrophobic interactions and hydrogen bonding, with hydrophobic/aromatic amino acid residues playing a critical role in binding [173,174]. Such interactions are associated with modifications in the sensory characteristics of food products.

## 8. Challenges and Future Perspectives

Studying the molecular interactions between wall materials and core compounds is essential for designing effective encapsulation systems. Despite significant advancements in encapsulation systems, several challenges remain in optimizing colloid–bioactive interactions for enhanced bioactive stability and bioaccessibility. To date, the majority of studies on encapsulation systems have been limited to laboratory-scale investigations. Factors such as the structural complexity and variability of biopolymers and environmental conditions limit the reproducibility and scalability of encapsulation systems. Although spectroscopic and microscopic techniques offer significant insights, the molecular mechanisms underlying non-covalent and covalent interactions are not yet fully understood, especially under gastrointestinal conditions. In addition, high EE% does not always result in improved release characteristics or bioaccessibility.

Future research should focus on multi-scale characterization approaches that integrate advanced analytical techniques such as FTIR, fluorescence spectroscopy, TEM, and SAXS with computational modeling tools, including molecular docking, molecular dynamics, and machine learning, to unravel interaction mechanisms at both molecular and colloidal levels. Integrating these approaches with experimental data can enable the development of delivery systems tailored to specific functional and nutritional goals.

## 9. Conclusions

Various colloids and bioactive compounds can coexist within food matrices or carrier systems, and their molecular-level interactions are of critical importance. Interactions occurring at the molecular level result in changes in the structures of both reacting substances, enabling the formation of various complex structures. Although recent studies in this field have demonstrated the potential to explain the macro-level impacts of these interactions, the functionality and bioactivity of the resulting complexes remain uncertain. Since the interactions between colloids and bioactive components in any matrix are highly likely to occur under the influence of multiple parameters, comprehensive research into the binding mechanisms and possible structural changes is necessary. Colloid-bioactive interactions play an important role in food applications and carrier systems by influencing product quality, stability, texture, and health effects. Thus, by evaluating the chemical changes occurring at the micro level, the design of products with the desired texture, functionality, and quality can be achieved.

Although the interactions of protein- and polysaccharide-based colloidal systems with polyphenols and carotenoids have been widely investigated in recent studies, future research requires more comprehensive approaches that establish a stronger relationship between the interaction mechanism, matrix structure, and functional properties. In particular, there is limited understanding of how binding type and surface properties determine their behavior and biological effects during gastrointestinal digestion. Therefore, in subsequent studies, standardized model systems are needed to facilitate comparisons across different systems. Lastly, in the near future, computer-based studies such as molecular docking will become even more widespread, enabling the achievement of desired macroscopic properties through targeted micro-scale alterations.

## Figures and Tables

**Table 1:** Covalent and non-covalent interactions of proteins and polyphenols.

Protein-Polyphenol Interactions	Interaction Type and Method	Characterization Techniques for Interactions	Structural Modification of Protein	Alterations in Functional Properties	Changes in Bioaccessibility and Antioxidant Activity of Polyphenols	References
Milk proteins-coffee polyphenols	Non-covalent interactions through mixing at pH 7	Fluorescence spectroscopy	Change in secondary structure of protein	Increased solubility interactions with coffee polyphenols.	n.i.	[23]
Chickpea protein isolate-epigallocatechin gallate	Covalent interactions through mixing at pH 9, 26 °C for 24 h, dialyzing, and lyophilization	Fourier Transform Infrared (FTIR) spectroscopy Fluorescence spectroscopy	Change in secondary structure of protein Increase in α-helix content and a decrease in β-sheet content	Approximately 100% increase in EAI and approximately 35% decrease in ESI after EGCG conjugation. Increase in solubility after EGCG conjugation for neutral and alkaline pH.	n.i.	[58]
Milk proteins-blackcurrant polyphenols	Non-covalent interactions through mixing at a pH of 6.5, 28 °C for one h and lyophilization	FTIR spectroscopy	Loosened structure increased the surface hydrophobicity of casein Increased β-sheet content of whey proteins	n.i.	Increased antioxidant activity (10–15%) and in vitro bioaccessibility (25–30%) with milk protein interactions	[49]
Chickpea protein isolate-naringenin	Non-covalent interactions through mixing at pH 7	FTIR spectroscopy	Slight shifts in the amide I and II bands Formation of electrostatic interactions	Increase in foaming capacity (FC) and foaming stability (FS) values of approximately 40% and 22%, respectively (at 0.1% NAR concentration). Decrease in FC and FS values with further increase in NAR concentration.	n.i.	[62]
Soy protein-hydroxytyrosol (HT)	Mixing SPI and HT solutions and freeze-drying	Fluorescence quenching FTIR spectroscopy	Alteration in secondary structure, decrease in α-helix content, and increase in β-sheet content	Increase in solubility and foaming values with increasing HT concentrations. Highest solubility (52.84%) and foaming value (93.51%) at 50 μmol/g and 30 μmol/g HT concentrations, respectively.	n.i.	[63]
Rice protein-legume polyphenols	Covalent interactions through mixing at 40 °C, and the pH of 8.0	FTIR spectroscopy	Formation of ester (C-O-C) and amine (C-N) bands.	Foaming capacity increased by 3 times, and foaming stability increased by 53.3%.	n.i.	[64]
Peanut protein extract-epigallocatechin gallate, chlorogenic acid	Alkali treatment method at pH 9 and lyophilization	Fluorescence quenching FTIR spectroscopy	Unfolding of protein, increasing the exposure of Tryptophan (Trp) residues with polyphenol	Increase in ESI value from 30 min to 80 min with PPE-EGCG and PPE-CA interactions. Increase in solubility of PPE from 67.8% to about 80% with PPE-EGCG complex and about 90% with PPE-EGCG complex.	n.i.	[65]
Goose liver protein- catechin, quercetin, and rutin	Non-covalent interactions through pH-shifting (from 11.5 to 7.0)	Fluorescence spectroscopy	Unfolding of protein structure Formation of hydrophobic interaction, hydrogen bonding, and van der Waals forces	Improved surface hydrophobicity. Larger particle size with rutin interaction due to protein aggregation. Emulsion droplet size reduced to 3.78 ± 0.27 µm upon quercetin interaction	n.i.	[44]
Soy protein isolate-chlorogenic acid	Covalent interactions through mixing at pH 9, 25 °C for 7 h	Fluorescence spectroscopy FTIR spectroscopy	The looser tertiary structure of protein. Altered secondary structure and protein conformation. Expose tyrosine and tryptophan residues to the hydrophilic environment. Covalent crosslinking	Increase in solubility of SPI with covalent crosslinking with CA. The highest solubility level (61.87%) at 0.05 g/g CA crosslinking. Decrease in surface hydrophobicity as 20.17%, 44.61%, and 56.13% for the samples with 0.002, 0.01, and 0.05 g/g CA crosslinking, respectively. Increase in gel hardness up to 0.01 g/g CA concentration.	n.i.	[66]
Soy protein-chlorogenic acid	Mixing of SPI and CA solutions at pH = 7	Fluorescence spectroscopy FTIR spectroscopy	Unfolded protein structure, Altered secondary structure, Decrease in α-helix and β-sheet with increase in β-turn and random coil.	Decrease in surface hydrophobicity with increasing CA concentration (up to 100 μmol/L). The highest solubility (47.77 %), ESI (40 min), foaming ability (3000%), and foaming stability (45%) with 80 μmol/g CA conjugation.	n.i.	[67]
Soybean protein isolate- apple polyphenols	Non-covalent interactions through mixing at a pH of 7	Circular dichroism (CD) spectroscopy	Altered secondary structure of proteins Transition from α-helix-to-random coil structure	Higher EAI (55.54 m^2^/g) and ESI (88.17%) with polyphenol interactions.	n.i.	[52]
Peanut protein-epigallocatechin gallate	Covalent interactions through mixing at 25 °C and freeze-drying	CD spectroscopy	Decrease in helix and random coil contents. Formation of hydrogen bonds.	Improved foaming properties.	n.i.	[68]
Pea protein isolate-chlorogenic acid, epigallocatechin gallate, resveratrol	Non-covalent interactions through mixing overnight at 4 °C, pH of 7	Fluorescence spectroscopy FTIR spectroscopy	Altered secondary structure of proteins Decreased α-helix and increased β-sheet content	Increased foaming capacity up to around 42.2–58.2%). The highest EAI (57.88 m^2^/g) achieved with EGCG interaction.	n.i.	[69]
Egg white protein-tea polyphenols	Non-covalent interactions through mixing at pH 7 °C, for two h, and lyophilization	CD spectroscopy Fluorescence spectroscopy	Modification of the protein structure at neutral pH, resulting in an increased ratio of α-helix and β-sheet, and consequently, the formation of hydrogen and hydrophobic interactions.	Improved emulsifying properties.	Increased antioxidant activity with increased polyphenol concentration. Higher antioxidant activity values at neutral pH (8.31–13.46 fold) than acidic pH (9.43–13.64 fold).	[11]
Casein, whey proteins, sea buckthorn polyphenols	Non-covalent interactions through incubation at pH 2.5 °C, for one h under pH 7	FTIR spectroscopy Fluorescence spectroscopy	Unfolded tertiary structure of protein Decrease in the α-helix, and an increase in β-sheet content. Exposure of hydrophobic groups	n.i.	Retained antioxidant activity through interactions with proteins. Higher in vitro bioaccessibility through casein and whey protein interactions (60.97–64.97%) than free polyphenolic extract (40.01%).	[36]
Soy protein isolate- epigallocatechin gallate	Covalent interactions through ultrasonication (at 300 W for 10 min, 2 s on/off)	Fluorescence spectroscopy FTIR spectroscopy	Altered protein structure Decreased α-helix content	n.i.	Highest DPPH (84.84%) and ABTS (88.89%) values with EGCG interaction compared to natural protein.	[70]
Soy protein isolate-tannic acid	Mixing SPI and TA solutions under alkali pH (9, 10, 11) and aerobic conditions	Fluorescence spectroscopy CD spectroscopy FTIR spectroscopy	Unfolded tertiary structure of protein Decrease in the α-helix, and an increase in β-sheet content, β-turn, and random coil.	Increase in gel hardness at pH of 11 to a certain TA concentration (117 μmol/g). The highest hardness level is 43.33 g.	n.i.	[71]
Bovine lactoferrin-procyanidin	Non-covalent interactions through mixing at pH 7	CD spectroscopy	Altered secondary structure of proteins Decrease in the α-helix due to disrupted hydrogen bondings.	The highest foamability (186%) and foam stability (65%) values were observed at the highest level of procyanidin complexation.	n.i.	[72]
Whey protein- epigallocatechin gallate, quercetin, apigenin, naringenin	Covalent interactions through free radical grafting at pH 7.2	Fluorescence spectroscopy FTIR spectroscopy	Altered secondary structure Decreased α-helixes and random coils	n.i.	Improved antioxidant activity with polyphenol interactions.	[6]
Whey protein-cholorogenic acid, epigallocatechin gallate	Non-covalent interactions through mixing in the dark at 25 °C for 60 min at pH 3.5 and 7	Fluorescence spectroscopy	Unfolded protein structure Distortion of the tertiary quaternary structure	n.i.	Increased reducing capacity and FRAP values with increasing phenolic concentration No significant change in ORAC values	[73]

CD, Circular Dichroism; FTIR, Fourier Transform Infrared Spectroscopy; n.i, not indicated.

**Table 2:** Effect of Starch-, Pectin- and Alginate-Based Carrier Mixtures on Carotenoid Encapsulation, Stability, and Release.

Carotene	Encapsulation Method	Carrier	Encapsulation/Loading Capacity	Physicochemical Properties	Release Kinetics/Bioaccessibility	Reference
β-carotene	Aggregation: High-speed homogenization and stirring	Octenylsuccinated *Gastrodia elata* starch	↑ Aqueous solubility of β-carotene from 1.5 × 10^−6^ to 58.96	NMR: β-carotene located in inner domains, proton peaks disappear UV-Vis (λmax): red shift, H/J-type aggregate structure FTIR: all β-carotene peaks disappeared → fully encapsulated, stabilized via hydrogen bonding XRD: Amorphous → stable aggregates ↓ Contact angle&Surface tension: Tight hydrophobic domain	Release profile (SGF/SIF): <15% in SGF at 120 min, slow increase in SIF Release kinetics: Weibull b = 1.476 → complex, super case-II transport Korsmeyer-Peppas *n* = 0.9858 → relaxation-dominated ↑ Bioaccessibility: 31.4%	[88]
β-carotene	Spray-drying: high-speed shear and homogenization	Octenyl succinic anhydride modified starch with tea saponins	↑ EE and LC ↓ Particle size and ζ-potential via formation of OSA-starch-tea saponin complexes	↓ Surface tension via tea saponin level FTIR: most β-carotene peaks disappeared → fully encapsulated, stabilized via hydrogen bonding XRD: Amorphous structure (low relative crystallinity 2.61%) with high tea saponin level	↑ Bioaccessibility: from 2.73 to 15.97%	[92]
Canthaxanthin	Freeze-drying, after ultrasonication (2–10 min, 200 W, 20 kHz)	V amylose (DP 311) + maltodextrin	Optimum: 20 mg canthaxanthin + 400 mg V-amylose → Canthaxanthin recovery ≈ 90%	TEM: Resemble spherical or oval shape after ultrasonication XRD: Fully amorphous → canthaxanthin loses crystallinity DSC: Canthaxanthin peaks disappeared → fully encapsulated ↑ Antioxidant activity: DPPH and ABTS	n.i.	[89]
β-carotene	Emulsification with ultrasonication	Mandarin peel pectin	EE: 78.7–87.4% ζ-potential: −25.7 to −27.1 mV after β-carotene loading	↑ Viscosity → higher emulsion stability and reduced β-carotene degradation	Bioaccessibility range: 29.20–62.97% according to oil type, highest for olive oil	[33]
β-Carotene	Pickering emulsion	Pectin	↑ Pectin concentration (0.5–3%): ↓ Creaming index (more stable), ↓ Droplet size	Thermal stability (TGA): Three-phase mass loss: 40–100 °C (loosely bound water, 51.7%), 200–310 °C (bound water, 11.59%), 330–430 °C (oil decomposition) FTIR-ATR: No new chemical bonds formed Rheology: Non-Newtonian, viscoelastic behavior	↑ Bioaccessibility: from 8.7 to 37.76%	[95]
Carotenoids from orange peel	Emulsion and gelation	Sodium alginate + CaCl2	Encapsulation yield: 92.3% EE: 89.5%	Optical microscope: Bead size: ~0.78 mm diameter, spherical ↑ Alginate concentration: I↑viscosity of continuous phase → restricted droplet movement and improved emulsion stability FTIR: Physical entrapment of oil in Ca-alginate beads; minimal chemical interaction Antioxidant activity (IC50): ~17 mg/mL before and after encapsulation, no significant change reported.	n.i.	[96]
Sea buckthorn pomace carotene extract:	Emulsification and ionic gelation	Sodium alginate	EE: 98.4%	SEM/RCM-Vis: Spherical, mean diameter ≈ 700 ± 50 µm; rough outer surface with cracks Fluorescence distribution: Carotenoids evenly dispersed; higher density near bead surface Storage stability for 30 days: Zeaxanthin → 46–50% retention Zeaxanthin esters → 66–79% retention (more stable than free xanthophylls) Carotenes → 54–72% retention	↑ Bioaccessibility: 42.1% Negligible release in SGF (surface carotenoids only); controlled release and micellization in SIF	[97]
Lycopene and β-carotene	Ionotropic gelation	Sodium alginate + CaCl_2_	Encapsulation yield: 86.3% EE: 82.6%	FT-IR: New broad O–H band Raman: ↓ Peak intensity	Total carotenoid retention after digestion:83.3%	[98]
Lutein	Hydrogelation	Sodium alginate + Ca^2+^-EGTA and D-gluconolactone	LC: 770.88 μg/g EE: 90% for nanoparticles, 99.39% for hydrogel ↑ Ca^2+^ concentration: ↑ Crosslinking density → better nanoparticle diffusion and higher EE	DSC: Disappearance of lutein melting peak → lutein exists in amorphous form FTIR: Carboxyl peaks red-shifted → stronger Ca2+–alginate interaction; confirms efficient encapsulation	After digestion: Higher Ca^2+^ → stronger network, slower diffusion and reduced release Lutein release: Increases then decreases for 4–20 mM Ca^2+^; max at 7.5 mM Ca^2+^ (71%)	[99]

DSC, Differential Scanning Calorimetry; EE, Encapsulation efficiency; EGTA, Ethylenebis (oxyethylenenitrilo) tetraacetic acid; LC, Loading capacity; NMR, Nuclear Magnetic Resonance; SEM, Scanning Electron Microscopy; SEM-RCM, Scanning Electron Microscopy–Reflectance Confocal Microscopy; SGF, Simulated gastric fluid; SIF, Simulated intestinal Fluid; UV–Vis, Ultraviolet–Visible Spectroscopy; XRD, X-ray Diffraction; n.i: not indicated. The symbols ↓ and ↑ denote a decrease and an increase, respectively.

**Table 3:** Effect of gum- or cellulose-based carrier mixtures on carotenoid encapsulation, stability, and release.

Carotenoid	Encapsulation Method	Carrier	Encapsulation Outcomes	Physical and Morphological Characterization	Release Kinetics/Bioaccessibility of Carotenoid	Reference
Lycopene-rich extract from red guava	Nanoprecipitation/high-shear homogenization	Cetylated cashew gum ± fucan	Particle Size: 189 → 251 nm EE: 8–13% for Cetylated cashew gum to ~60% with fucan	Thermal and storage stability: Stable for 90 days at 4 °C; retained ~60% of the lycopene content. FTIR: FTIR Spectra: Shifts in C=O and S=O bands TEM/AFM/NTA: spherical with smooth surfaces ↑ Antioxidant Activity (ABTS/ORAC)	n.i.	[101]
β-Carotene	Spray-drying	Maltodextrin + acacia gum or mesquite gum	Encapsulation yield: Acacia gum → up to 85%; mesquite gum → 69%	Antioxidant activity (TEAC/g): Acacia gum → 49–61 mesquite gum → 22–31 Carotenoid Content (μg/g): Acacia gum → 23–74; mesquite gum → 16–20	n.i.	[102]
Lycopene	Emulsion: high-pressure homogenization and spray drying	Basil seed gum	Optimized Encapsulation Conditions: ≈19.5% gum level → EE: 86.78%, Encapsulation yield: 54.94% PDI < 0.30 (uniform dispersion); ζ-potential: −21.37 mV	Antioxidant Activity (DPPH): Initially increased with gum level but later declined at high carrier concentrations	n.i.	[87]
β-Carotene	Emulsification:	Xanthan gum, guar gum	EE: 87.2%	↑ Chemical stability	n.i.	[103]
β-carotene	Emulsification: high-speed shear and ultrasonic emulsification	High acyl gellan gum	Emulsification yield: gradual increase via gum concentration (0.05–0.2%), highest 84.4% at 0.175% Mean particle size: 4–7.2 µm ζ-potential: between −59.0 and −52.2 mV	Rheology: low gum concentration → near Newtonian; higher gum concentration → pseudoplastic (shear-thinning) Emulsion stability (DBS): best at 0.175% HA (minimal change in back-scattering) ↑ Thermal, NaCl, pH stability	↑ Bioaccessibility: 18–26.6%	[104]
β-carotene	Pickering emulsion: high-shear homogenization + high-pressure homogenization	Peach gum polysaccharide: MC, YQG, ZP11, TW-20	EE: 71.92–89.08%	SEM: Homogeneous, densely distributed, regular	Degradation Kinetics: first-order reaction kinetics, the lowest β-carotene loss for YQG; Half-life: MC 24.8 d, YQG 32.3 d, ZP11 23.5 d, TW-20 27.3 d	[90]
Natural palm mixed-carotene complex	Pickering emulsion	Nanofibrillated cellulose	EE: 87–89.80% ↑ Particle size: 73.67 → 94.73 via cellulose concentration (0.2–1%) ζ-potential: <−30 mV	n.i.	n.i.	[95]
Lycopene	Pickering emulsion	Cellulose nanofiber from papaya peel	n.i.	n.i.	Higher oil fraction → slower intestinal release → better controlled-release Release percentage in SGF: 9.8–15% and in SIF 68.2–43.2 via oil concentration (10→60%)	[86]
β-carotene	Pickering emulsion	Cellulose nanofibers	EE: decreased from 94.24% at 25 °C to 57.23% at 75 °C	n.i.	↑ Bioaccessibility: from 35.62 to 59.74	[91]
Astaxanthin	Cold application gel	Hydroxypropyl methylcellulose, types: K1500, K250, K4M, E5	Optimal HPMC-K4M dosage: 0.2 g or 107.50%	Antioxidant activity (DPPH): highest for K4M	K4M: highest in vitro release	[105]

EE, Encapsulation efficiency; LC, Loading capacity; SGF, Simulated gastric fluid; SIF, Simulated intestinal Fluid; n.i: not indicated. The symbols ↓ and ↑ denote a decrease and an increase, respectively.

**Table 4:** Effect of Various Polysaccharide-Based Carrier Mixtures on Carotenoid Encapsulation, Stability, and Release.

Carotenoid	Encapsulation Method	Carrier	Encapsulation Outcomes	Physical and Morphological Characterization	Release Kinetics/Bioaccessibility of Carotenoid	Reference
Lutein	Emulsion: Gelation and centrifugal washing	Curdlan ± Sodium Alginate	Optimumencapsulation: Curdlan/Sodium Alginate ratio → 1.97/1 Water-to-oil volume ratio →1:2 Acetic acid addition → 0.49% ↑ EE, LC, particle size for complex system Curdlan ± Sodium Alginate Emulsion: EE: 80.19% LC: 11.60%	SEM: Smooth and less porous XRD: Sharp crystalline peaks of free lutein disappeared, encapsulation in an amorphous state Reology: ↑ G′ and G″ values ↑ Particle size ↑Storage/light/thermal stability DSC: ↑ Tg	Lowest SGF release (24.6%) and highest cumulative release (81.97%) Degradation kinetics: Longest half-lives, highest antioxidant activity	[114]
β-Carotene:	Liposome	Egg yolk phosphatidylcholine + cholesterol + chitosan ± pectin	↑ EE via pectin addition from 82.34 to 84.7% ↑ Mean particle size, polydispersity index, and ζ via chitosan ± pectin Appropriate concentration for pectin: 0.06% for balanced size, charge, and EE	DSC:↑ T_m_: 122.46 °C for chitosan, P-C-L: 131.81 °C) for chtiosan + pectin TGA/DTG: ↑ Thermal stability	Chitosan + pectin liposome: Longer-term controlled release in SGF and SIF Diffusion + swell particles shrink and become more homogeneously dispersed	[111]
Astaxanthin	Ionotropic gelation	Pectin + sodium alginate + chitosan in oleoresin	↑ EE: ≈87% ↓ Oil leakage:	Highly spherical via alginate ↓ Lipid oxidation (PV and p-AV) Thermal stability for astaxanthin retention (65 °C stress test): Pectin + chitosan: best retention zero-order kinetics Pectin and pectin + alginate + chitosan: faster degradation, two-slope kinetics	No release in SGF SIF: Pectin and pectin + chitosan: ~46–48% release; pectin + alginate + chitosan: ~58% release	[113]
Lutein	Ionic gelation	Chitosan (1 mg/mL) and sodium alginate (0.5 mg/mL)	Mean particle size: 98 nm Polydispersity Index: 0.27 ζ: +38 mV	SEM/TEM: Smooth, discrete, spherical	Male Wistar rats: ↑ Cmax and AUC, ↑ Mean residence time, prolonged half-life, and slower plasma clearance	[112]
Lutein	Complex coacervation	Alginate + low-molecular-weight chitosan	EE. 98–99.93% ζ-potential: ≈11 mV	Thermal stability: TGA/DSC: ↑ Thermal stability, endothermic lutein peaks disappeared XRD: Crystalline peaks of lutein disappeared → amorphous form in alginate + chitosan complexes ↑ Thermal and Light stability	n.i.	[109]
Astaxanthin	Pickering emulsion	Chitosan + Guar gum	↓ Droplet size via pH (3→6) Most stable: ↑ Creaming index	FTIR: intermolecular H-bonding Droplets homogeneous, no flocculation → high stability Rheology: G′ > G′′ → elastic structure ↑ Storage and Oxidative stability	Chemical astaxanthin retention: chitosan + guar gum (85.9%) > chitosan (77.85%) > Tween 80 (66.12%)	[110]
β-Carotene:	Host–guest inclusion + coating	2-Hydroxypropyl-β-cyclodextrin + High- or low-metoxyl pectin or pectic acid	↓ Particle size and ζ-potential: The most stable for high-methoxy pectin after 4 weeks of storage at 4 °C	FTIR: New hydrogen bonding in ternary complex DSC: ↑ Thermal stability, highest for high-methoxy pectin ↑ Thermal stability (60 and 95 °C) Pectin type effect: high-methoxy pectin > low-methoxy pectin > pectic acid	n.i.	[115]
β-carotene: (20% *w*/*w*)	Gelation	Yam starch + guar gum, xanthan gum, carrageenan gum, or their combinations	n.i.	FTIR: Shifts in O–H and C–H stretching indicated stronger hydrogen bonding with chitosan and weaker with gum arabic XRD: ↓ Crystallinity for all hydrocolloids; lowest for chitosan, highest for guar gum and carrageenan gum.	n.i.	[117]
Lycopene: 0.5, 1.0, and 1.5	Emulsion-based polyelectrolyte complex	Sodium alginate + CaCl_2_ + chitosan	EE: from ~53 to 86.85% Particle size: 148.7–152.8 nm	↑ DPPH antioxidant activity SEM: quasi-spherical, clustered particles	n.i.	[118]
Astaxanthin: 0.5 g	Tertiary emulsion: layer-by-layer self-assembly	Soy lecithin + chitosan + sodium alginate	LC: ≈ 0.56% EE > 90% ζ-potential: primary: −11.6 → −8.25 mV; secondary: 29.5 → 24.6 mV; tertiary: −22.7 → −18.4 mV	n.i.	Slower, controlled in vitro release ↑ Bio-accessibility: 18% in SGF → 69–70% in SIF vs. suspension (0.2–25%).	[116]

n.i: not indicated. The symbols ↓ and ↑ denote a decrease and an increase, respectively.

**Table 5:** Summary of recent studies on protein–polysaccharide-based delivery systems for improving bioactive encapsulation and bioaccessibility.

Wall Material	Core Material	Encapsulation Method	Key Findings	Reference
Chickpea protein isolate (CPI) Citrus pectin (CP)	Curcumin	Emulsion formation by high-pressure homogenization with CPI–CP conjugates	Emulsion stabilized with CPI-CP exhibited higher EE% (88.79) compared to CPI alone (84.30%). CPI-CP emulsions provided better curcumin stability during storage at room temperature, as well as under environmental stressors. Compared to CPI emulsions, CPI-CP had lower release of FFA in SGF and higher in SIF. It was found that CPI-CP emulsions had higher curcumin bioaccessibility, which was 85.64%.	[125]
Quinoa protein (QP) Inulin (INU), sodium alginate (SA), fucoidan (FU), and dextran (DX)	Curcumin	Emulsion gel formation by glucono delta-lactone induced gelation using QP-polysaccharide mixtures	The addition of polysaccharides enhanced the EE% of curcumin compared to emulsions stabilized by QP alone. QP-SA emulgels exhibited a lower release of curcumin in SGF and a slower sustained release in SIF. Compared to QP, QP-INU, and QP-FU emulgels, QP-DS and QP-SA gels exhibited a controlled release profile with lower FFA release in initial gastric conditions and higher release in SIF. Incorporation of FU increased the curcumin bioavailability. However, QP-DX and QP-SA emulgels had lower EE% compared to QP and QP-FU, potentially due to undigested oil droplets.	[127]
QP)-DX conjugates	Curcumin	Nanoemulsion formation by high-pressure microfluidization using QP–DX conjugates as stabilizers	Nanoemulsions stabilized by QP-DX conjugates exhibited significantly higher EE% (74.5%) compared to QP-DX complexes (67.3%). A higher release of FFA occurred during the in vitro digestion of nanoemulsions stabilized by QP-DX conjugates compared to QP-DX complexes. Nanoemulsions stabilized by QP-DX conjugates provided higher curcumin bioaccessibility (30.38%) compared to QP-DX complexes (22.86%).	[128]
Mung bean protein (MBP) Sugar beet pectin (SBP)	Riboflavin	MBP–SBP hydrogel formation via laccase-induced crosslinking	The increase in SBP resulted in increased EE% in composite hydrogels. Composite hydrogels showed better stability in both SGF and SIF compared to MBP hydrogel. Higher concentrations of SBP resulted in increased riboflavin release in SIF. Higher concentrations of SBP provided better retention of riboflavin after 28 days of storage. Composite hydrogels exhibited higher riboflavin bioaccessibility.	[123]
SPI Soy hull polysaccharide	*Lactobacillus plantarum*	HIPE formation by homogenization of SPI–SHP mixtures with soybean oil	As the SHP concentrations increased in HIPEs, *L. plantarum* viability gradually increased in SGF. HIPEs with the highest SHP concentration provided the highest delivery efficiency in SIF, which was 71.1%.	[129]
SPI Xanthan gum (XG)	Quercetin	Pickering emulsion formation by homogenization of SPI–XG mixtures with corn oil	In CLSM images, it was observed that oil droplet sizes of PEs decreased as the XG concentration increased, which resulted in a higher contact area with lipase. In parallel to CLSM observations, PEs with the highest XG concentration exhibited increased FFA release. SPI-XG PEs showed better resistance in SGF compared to SPI alone, which provided lower quercetin release. The release in SIF and bioaccessibility of quercetin increased as the XG concentration increased, potentially due to increased surface area.	[130]
SPI-peach gum conjugate	Lycopene	Microparticle formation by spray drying using SPI–PG Maillard conjugates	The highest EE% was tested for the spray-dried powders with the highest wall material concentration, which was 90.82%. During the 21 days of storage at −20, 4, 25, and 50 °C, non-encapsulated lycopene rapidly degraded at all temperatures, while SPI-PG conjugate microparticles enhanced the retention of lycopene. Lycopene was mostly released in SIF from SPI-PG microparticles, while free lycopene showed insignificant release throughout in vitro gastrointestinal digestion.	[124]
PPI-DX conjugates	Astaxanthin	Emulsion formation by high-pressure homogenization using PPI–DX complexes and conjugates	Compared to PPI-DX complexes, PPI-DX conjugate stabilized nanoemulsions provided higher astaxanthin bioavailability. After 28 days of storage at 4 °C, PPI-DX conjugate stabilized nanoemulsions exhibited higher astaxanthin retention (above 70%).	[105]
SPI Soy hull polysaccharide (SHP)	Lutein	Liposome formation by thin-film evaporation–sonication and incorporation into SPI–SHP hydrogel matrix	SPI-SHP gels had a higher EE% compared to liposomes alone. The EE% of liposomal gels increased with higher SHP concentrations. During in vitro gastric digestion, liposomes released 44.44% of lutein, while SPI-SHP liposomal gels provided better protection with lower release. However, in SIF, SPI-SHP gels exhibited higher lutein release. The bioavailability of lutein in SPI-SHP gels ranged between 20 and 40%, which was higher than that of liposomes alone.	[126]

CLSM, confocal laser scanning microscope; CP, citrus pectin; CPI, chickpea protein isolate; DX, dextran; EE, encapsulation efficiency; FFA, free fatty acids; FU, fucoidan; GDL, glucono delta-lactone; HIPE, high internal phase emulsion; INU, inulin; PPI, pea protein isolate; PG, peach gum; QP, quinoa protein; SA, sodium alginate; SBP, sugar beet pectin; SHP, soy hull polysaccharide; SGF, simulated gastric fluid; SIF, simulated intestinal fluid; SPI, soy protein isolate; XG, xanthan gum.

## Data Availability

No new data were created or analyzed in this study. Data sharing is not applicable to this article.
